# QuantWorm: A Comprehensive Software Package for *Caenorhabditis elegans* Phenotypic Assays

**DOI:** 10.1371/journal.pone.0084830

**Published:** 2014-01-08

**Authors:** Sang-Kyu Jung, Boanerges Aleman-Meza, Celeste Riepe, Weiwei Zhong

**Affiliations:** Department of Biochemistry and Cell Biology, Rice University, Houston, Texas, United States of America; McGill University, Canada

## Abstract

Phenotypic assays are crucial in genetics; however, traditional methods that rely on human observation are unsuitable for quantitative, large-scale experiments. Furthermore, there is an increasing need for comprehensive analyses of multiple phenotypes to provide multidimensional information. Here we developed an automated, high-throughput computer imaging system for quantifying multiple *Caenorhabditis elegans* phenotypes. Our imaging system is composed of a microscope equipped with a digital camera and a motorized stage connected to a computer running the QuantWorm software package. Currently, the software package contains one data acquisition module and four image analysis programs: WormLifespan, WormLocomotion, WormLength, and WormEgg. The data acquisition module collects images and videos. The WormLifespan software counts the number of moving worms by using two time-lapse images; the WormLocomotion software computes the velocity of moving worms; the WormLength software measures worm body size; and the WormEgg software counts the number of eggs. To evaluate the performance of our software, we compared the results of our software with manual measurements. We then demonstrated the application of the QuantWorm software in a drug assay and a genetic assay. Overall, the QuantWorm software provided accurate measurements at a high speed. Software source code, executable programs, and sample images are available at www.quantworm.org. Our software package has several advantages over current imaging systems for *C. elegans*. It is an all-in-one package for quantifying multiple phenotypes. The QuantWorm software is written in Java and its source code is freely available, so it does not require use of commercial software or libraries. It can be run on multiple platforms and easily customized to cope with new methods and requirements.

## Introduction

Phenotypic assays are crucial in genetics. However, traditional methods relying on manual analysis are a bottleneck in large-scale quantitative experiments. As an alternative, automated high-throughput imaging systems have emerged. Several research groups have developed high-throughput imaging systems for *Caenorhabditis elegans* phenotypic assays and have demonstrated these systems to be highly successful in their applications [1], [2], [3], [4], [5], [6]. For example, various worm trackers [2], [3], [7], [8] can analyze worm locomotion; the Lifespan Machine [5] and WormScan [1] measure worm lifespan; some systems count the number of worm embryos [9] or focus on analyzing worm phenotypes in liquid culture [4], [10]. While these systems are useful for their specific applications, they fall short in screens when multiple phenotypes are analyzed. Further, most of these systems require commercial software such as a LabView Runtime license, MATLAB, or BioApplication [2], [3], [7], [8], [10]. Thus the goal of this study was to develop an automatic phenotyping system that (1) enables a comprehensive analysis of multiple phenotypes; (2) allows full handling of hardware and software and its source code; (3) has no dependency on commercial software; (4) runs on multiple platforms; and (5) enables sustainable system development to accommodate new analysis.

We developed the QuantWorm system that can analyze *C. elegans* lifespan, locomotion, body size, and egg laying phenotypes. The software was developed in Java, a free, cross-platform programming language, and can be installed and run on both Mac and Windows computers. The software can be used for free, as it does not require any commercial software. The QuantWorm source code is freely available at http://www.quantworm.org/, making it easy to modify and customize the system for new applications.

We demonstrated the utility of the QuantWorm system in two case studies: a drug assay and a genetic assay. First, we used our system to study the effect of celastrol on *C. elegans* lifespan. Celastrol is a natural triterpenoid purified from the root extract of the medicinal plant *Tripterygium wilfordii*. Celastrol has shown some promising anti-oxidant, anti-inflammatory, and anti-cancer activities [11], [12], [13], [14], [15]. Consequently, we hypothesized that celastrol might increase lifespan.

In our second case study, we quantified multiple phenotypes of *C. elegans* mutants in the Gαq/EGL-30 signaling pathway. The Gαq/EGL-30 pathway is a signal transduction pathway that influences lifespan, oxidative stress, immunity, locomotion, and egg laying, as mutants in this pathway have shown phenotypes in these processes [16], [17], [18], [19], [20], [21]. We decided to systematically quantify four phenotypes (lifespan, locomotion, body size, and egg laying rate) of mutants of five pathway components (TAX-6, EAT-16, Gαq/EGL-30, PLCβ/EGL-8, and UNC-73/Trio RhoGEF) using the QuantWorm system.

## Methods

### Nematodes

Nematodes were cultured on NGM (Nematode Growth Medium) agar according to standard protocols [22]. To obtain synchronized populations of *C. elegans* worms, eggs were harvested by bleaching gravid worms with a diluted alkaline hypochlorite solution [23]. The solution contained 0.5 M NaOH and 1% sodium hypochlorite from household bleach. The eggs were incubated overnight in M9 buffer supplemented with cholesterol at 5 mg/L. Hatched L1 larvae were transferred to NGM plates seeded with *E. coli* (OP50) and grown at 20°C. Assays were conducted using wild-type (Bristol N2), PS2960 *eat-16(sy438)*, MT1083 *egl-8(n488)*, PS3202 *egl-30(ad809)*, PR675 *tax-6(p675)*, and KG1278 *unc-73(ce362)*. PS2960 and PS3202 were obtained from the Sternberg lab. All other strains were obtained from the Caenorhabditis Genetics Center (CGC).

### Microscopy

The microscopy configuration in the QuantWorm system consists of a dissecting microscope (Unitron), a motorized stage and stage controller (Prior), and a Firewire camera (Unibrain Fire-i). The camera and stage controller are connected to the same computer.

### Phenotypic Assays

#### Lifespan assay

Six-well NGM plates were seeded with 70 µL of fresh overnight culture of the OP50 bacteria per well and incubated overnight at room temperature before use. Approximately 70 hatched L1 larvae in solution were dropped onto each well. At the L4 larval stage, the plates were dosed with FUdR at 25 µmol/L agar to prevent eggs from hatching. In lifespan assays with celastrol, the worms were dosed with celastrol at 7 µmol/L agar every other day from the L1 larval stage until all of the worms were dead.

Wells were imaged daily with our WormScanner program until all worms died. Two scans of twelve images (3×4 images of 640×480 pixels) per well were taken at two minute time intervals. These tiled images were assembled into a single large image of the entire well. We also conducted a traditional lifespan assay using a basic light microscope. A worm was scored as dead if it failed to respond to a touch with a platinum wire. Lifespan was scored with the first day of adulthood as day 0.

#### Locomotion assay

Two locomotion assays were conducted to compare the locomotion of different mutants with and without food. The first, a locomotion assay with food, was conducted in conjunction with the lifespan assay. 30-second videos (640×480 pixels) were collected for each well in the lifespan assay, when the animals were first day adults. Worms captured on videos were within the circular *E. coli* lawn. For the second assay, a locomotion assay without food, ∼100 worms were grown in seeded 6-well plates until they were first day adults. The worms were rinsed with cold S-basal solution three times and then transferred to unseeded 6-well NGM plates. Approximately 20 minutes after worms were dropped onto each well, videos were recorded for 30 seconds for each well.

#### Body size assay

Synchronized L1 larvae were dropped onto seeded 6-well plates and grown at 20°C. One day after the L4 larval stage, worms were collected from each well with M9 solution and transferred to unseeded 6-well plates. The adult worms were then killed by adding 20 µL of 1 M sodium azide into each well. During each scan, 130 tiled images were taken of each well.

#### Egg laying assay

Synchronized worms were grown in seeded 6-well plates until 28 hours after the L4 stage. The plates were washed with M9 buffer, and worm solution containing ∼10 adult worms was dropped onto 24-well plates with NGM wells seeded with 10 µL OP50 bacteria. The worms were incubated for 90 minutes at 20°C before 15 µL 1 M sodium azide was added into each well to kill the worms and bacteria. The plates were then scanned.

### Data Analysis

In the lifespan assay, the number of detected living worms should be equal to or greater than the number detected in later days. However, our image analysis method does not always guarantee this because more worms may be detected in later days. Thus a step wise decrease filter was applied to the raw data where if the number of living worms at day *t-1* is smaller than that at day *t*, we set the number of living worms at day *t-1* to the same as the number of living worms at day *t*. Unless otherwise specified, all measurements of the mean lifespan, worm speed, body length, and egg laying rate are given as the mean ± standard deviation from at least two independent experiments conducted at different days. At least triplicate wells were used within a given experiment.

## Results

### The QuantWorm Phenotyping System

**Figure 1:**
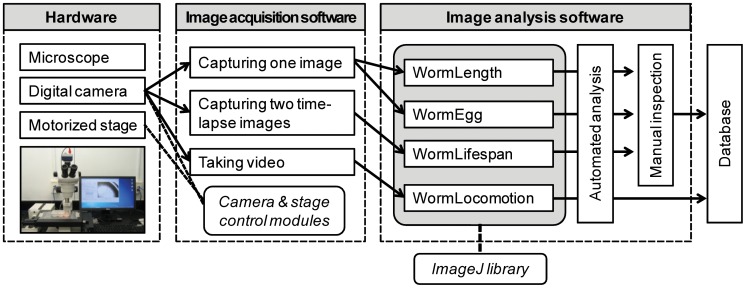
The QuantWorm imaging system. Our imaging system consists of imaging hardware and the QuantWorm software package. The hardware is composed of a microscope equipped with a digital camera and a motorized stage. The image acquisition software is used to control the stage and take images and videos. Four image analysis software programs are used to analyze body size, lifespan, egg laying, and locomotion from the images or videos. Once the software finishes the fully automated image analysis, a user can correct errors in the manual inspection window. Both native image processing algorithms and ImageJ API/library are used to process images.

The hardware of the QuantWorm system is composed of a microscope equipped with a digital camera and motorized stage. The software package in this system contains one image acquisition software (WormScanner) and four image analysis programs (WormLifespan, WormLocomotion, WormLength, and WormEgg) for lifespan, locomotion, body size, and egg laying assays (Figure 1). For image processing, the QuantWorm software uses the ImageJ API/library together with our native image processing libraries. Software source code, executable software programs and sample images are available at http://www.quantworm.org/. The image analysis software programs provide a similar 3-step process. ‘Image Processing’ conducts batch processing to automatically analyze multiple images/videos. ‘Manual Inspection’ enables a user to examine image analysis and conduct manual correction if needed. ‘Print Report’ creates text files detailing the phenotypic measurements.

#### WormScanner

The WormScanner is an automated image and video acquisition software that controls a motorized stage (Figure 1). In the image scanning mode, the software takes multiple tiled images of a Petri dish or individual wells of a multi-well plate. In the video recording mode, the WormScanner takes videos with specified length at given locations. For the lifespan assay, the software scans a plate twice to create time-lapse images.

#### WormLifespan

**Figure 2:**
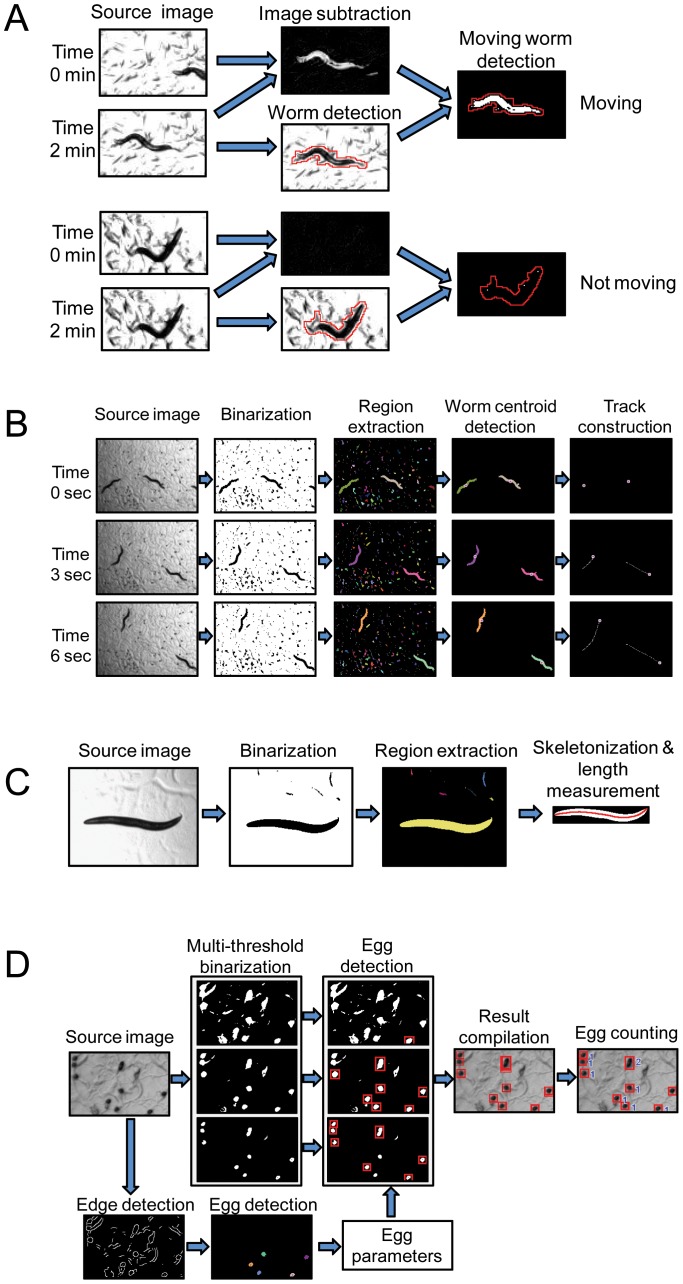
QuantWorm image processing algorithms. (A) WormLifespan. Two time-lapse images are obtained, and a differential image is created by subtracting the second time-lapse image from the first time-lapse image. Independently, individual worms are detected in the second image and are defined as region of interest (ROI). Worm movement is determined by counting the number of white pixels. (B) WormLocomotion. Image frames from videos are binarized and region-extracted to detect objects. Worms are indentified by analyzing the morphology of detected objects. An individual worm track is constructed by connecting all centroid points of a moving worm. (C) WormLength. Source image is binarized, and worms are detected by region extraction and shape analysis. Once a worm object is identified, a skeleton curve is created through the middle of the worm. The length of the worm is calculated by measuring the length of the skeleton curve (D) WormEgg. Single eggs are detected by applying edge detection, gap filling, flood filling, and morphology analysis. Egg detection parameters are determined by analyzing the detected single eggs. Multi-thresholding binarization is applied to create multiple binary images from which eggs are detected. Results are compiled to conduct clustering to identify highly probable eggs and remove duplicate findings.

WormLifespan counts moving worms from two consecutive images taken with a certain time interval (for example 2 minutes). Since living worms move or change their body shape, it is possible to detect such moving worms by comparing time-lapse images (Figure 2A). WormLifespan automatically identifies moving worms by analyzing the differential image created by subtracting the first time-lapse image from the second time-lapse image. The differential image is then binarized so that pixels that changed values are highlighted as white. A detected object in the second time-lapse image is a moving worm if its size is within a limit and if a certain number of white pixels are found in the binarized differential image. In addition, missing moving worms bound to large dark objects are detected by region labeling of the subtracted binary image. The software conducts parameter-free binarization using adaptive local thresholding [24] to analyze a wide range of plate images with different brightness/contrast levels or uneven illumination across a plate. One of the unique strengths of WormLifespan is that all detection algorithms used in the software have been optimized to detect moving worms even if an agar plate is shadowed or contains dark particles.

#### WormLocomotion

The WormLocomotion software conducts fully automated video analysis to compute the velocity of moving worms. Paths of moving worms are constructed by tracking individual worms based on their centroid and size in the region-labeled image (Figure 2B). Since flickering of the video sequences can introduce random positional noise into the centroid, all locomotion trails are smoothed by the Bezier path-fitting algorithm. The software keeps track of every individual moving worm until it collides with others or touches the boundary of image. The mean velocity is computed by dividing the distance traveled by the time elapsed. Any object whose trail is confined in a tiny bounding box is detected as a non-moving object and excluded from the locomotion analysis. The WormLocomotion software outputs results in several formats: (1) a binary file containing raw data of detected tracks; (2) two summary text files containing data of the average velocity and individual velocities of detected tracks; (3) two histograms showing distribution of worm velocities and cumulative probability distribution of worm velocities; and (4) an image file showing the last video frame with detected tracks in color.

#### WormLength

WormLength is an image analysis software that measures worm body size. For image processing, the source image is converted into a region-labeled binary image by applying adaptive thresholding and region labeling (Figure 2C). Any objects that fall outside set parameters (area and bounding box size) are excluded from further analysis. The software then computes a skeleton curve running through the middle of each valid worm. Only a worm having a single skeleton curve from head to tail without any branches is considered as a valid finding for body size measurement. The body length is calculated by summing up the distance between pixels while tracing the skeleton curve in the horizontal, vertical, or diagonal directions. The software removes false positive detection by determining the size, length, and fatness of detected objects. The presence of background particles or eggs often distorts skeleton curves; however, distorted skeleton curves are easily detected during manual inspection and removed from the data set.

#### WormEgg

The software counts eggs from a single image. Compared with the detection of adult worms, the detection of eggs is more challenging since worm tracks interfere with the image analysis. We thus developed a parameter-free detection algorithm, which applies multiple thresholds that create multiple binary images from a single source image (Figure 2D). We utilized the facts that most eggs dominantly appeared in several binary images and that obscured eggs could be detected in a certain subset of binary images.

The egg detection algorithm consists of five major steps: (1) identifying single egg objects; (2) determining valid egg detection parameters; (3) applying multiple thresholds; (4) analyzing morphology of detected objects; and (5) removing duplicate findings. The purpose of the first step is to locate single egg objects (not aggregated eggs) whose morphology parameters are used for in-depth analysis in the following steps. Valid single egg objects can be detected by applying Canny edge detection, gap filling, region labeling, morphology analysis, and single egg object detection. In the second step, the software determines proper reference threshold values such as average gray value and size of valid eggs. The next step detects every egg by applying multiple thresholding. Multiple binary images (n ≥ 10) are created from a single source image using different threshold values (for example, from 30 to 230 gray value with a step of 10). Once binary images are created, region labeling and morphology analysis are conducted for every binary image. This multiple thresholding approach helps find hidden eggs that might not be detected using a typical single step binarization method. Since multiple thresholding generates many replicate findings, a clustering process is performed to identify unique eggs and eliminate replicates, based on the location and size of eggs and the total number of occurrences in binary images.

### Software Accuracy

**Figure 3:**
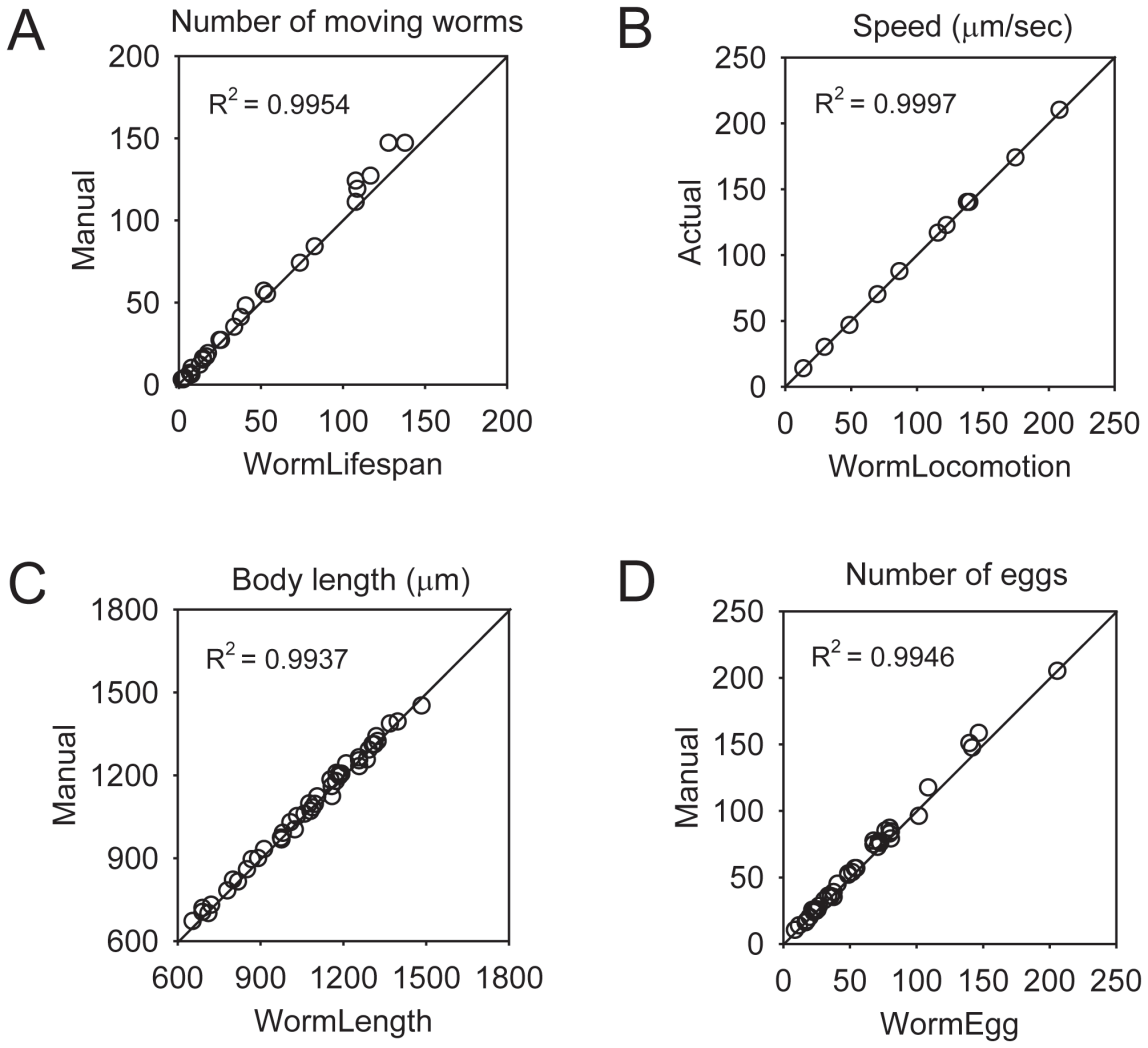
Performance of QuantWorm. (A) WormLifespan. Moving worms were manually counted by aspirating individual worms from a well under a basic light microscope after images were captured (*n* = 26 wells). (B) WormLocomotion. Worm simulation videos were created and then analyzed by the WormLocomotion software (*n* = 11 videos). (C) WormLength. In the manual method, worm length was manually measured from images using an Adobe Photoshop length measurement tool (*n* = 46 worms). (D) WormEgg. In the manual method, eggs were manually counted by aspirating eggs from a well after images were taken (*n* = 42 wells). The diagonal line represents the ideal case where the computer measurements equal the manual measurements.

To evaluate the accuracy of WormLifespan, we compared the result of image analysis using WormLifespan to the result of manual counting of living worms. In the manual counting method, the number of living worms was manually counted by aspirating individual worms under a microscope. From the direct comparison between the two methods, we found that the result of WormLifespan was highly correlated with that of the corresponding manual method with a correlation coefficient of R^2^ = 0.9954 (Figure 3A). The average difference in the worm count between the two methods was 3.7±5.12 worms. For six-well plates, the optimal condition is 0∼100 worms per well for our system. In populations greater than 100 worms, more worms crawled to the sides of the wells where they were shadowed from view and could not be detected by our software.

To test the accuracy of our locomotion software, worm simulation videos (Movie S1) that mimic sinusoidal movement of real worms were created using a simulation program and analyzed by WormLocomotion. The reason for analyzing the worm simulation videos was that manual measurement of the average velocity from actual worm videos was inconvenient and inaccurate. The simulation program, available at our QuantWorm website (http://www.quantworm.org/), rendered a series of black and white frame images containing multiple virtual worms. The locations of the virtual worms were designed to move straight forward to random directions at constant speeds (pixels/frame). A sine function was used to create a sinusoidal worm shape, and its amplitude and phase were modulated to simulate the worm shape. To evaluate the accuracy of WormLocomotion, worm velocities in the simulation videos were then compared with the average velocities analyzed by WormLocomotion. From the analysis, the average percent difference in the velocity between the two methods was 1.1±1.46%, indicating a high degree of analysis accuracy using WormLocomotion (Figure 3B).

To evaluate the accuracy of WormLength, we compared the result of image analysis using WormLength to the result of a manual method in which the body lengths of individual worms were manually measured using a length measurement tool in Adobe Photoshop. In the manual method, multiple straight lines were used to measure the entire length of any curved worm shape. The correlation coefficient and average percent difference in the body length between the two methods were R^2^ = 0.9937 and 1.3±0.93%, respectively (Figure 3C).

To evaluate the accuracy of WormEgg, we compared the result of analysis using WormEgg to the result of manual egg counting through a microscope. We found that the average difference in the egg count between the two methods was 2.6±2.95 eggs (Figure 3D). This result indicates that image analysis using WormEgg allowed an accurate measurement of egg count. It is worthy to mention that most eggs were found near and within the OP50 bacterial lawn in the center of the well in an agar plate, and eggs were hardly found in the dark side of the well near the wall. Consequently, the number of undetected, eggs hidden in the dark side of the well on the plate had a negligible effect on the result of our egg-laying assay.

### Case Study 1: Drug Assay

**Figure 4:**
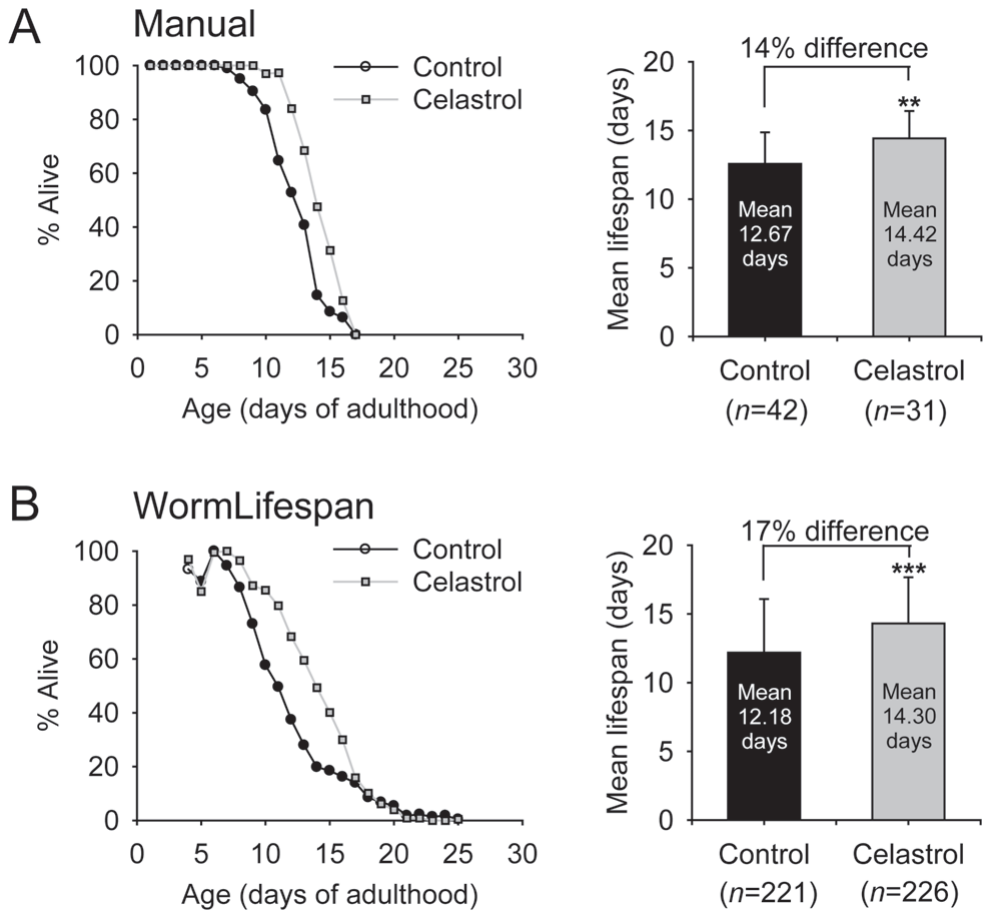
Celastrol increased lifespan. A lifespan assay was conducted using the manual method (A) or using the QuantWorm system (B). Worms were dosed every other day with 7 µmol/L agar celastrol or the solvent DMSO as a control. *n* represents the number of worms. **p<0.05; ***p<0.001; *p*-value by log rank test.

To assess the accuracy of WormLifespan software, we compared the results of a lifespan assay conducted by manually counting worms under a microscope with the results of an assay using the QuantWorm system. The data obtained using these two methods were highly consistent. In both methods, celastrol significantly increased the mean lifespan (Figure 4). In both the celastrol and the control groups, the mean lifespan measured by QuantWorm was very similar to that measured by the manual method with a percent difference less than 5%.

### Case Study 2: Genetic Assay

**Figure 5:**
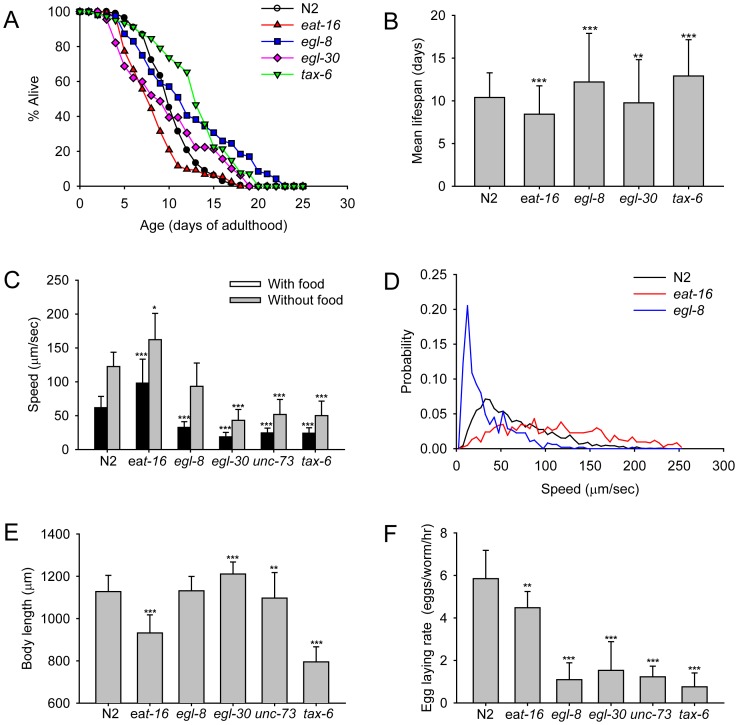
Phenotypes of Gαq pathway mutants. (A) Survival curves. The results are from two independent experiments. At least triplicate wells were used (*n* >180 worms for each strain). (B) Mean lifespan. (C) Worm speed measured as the sum of average worm velocities in individual videos divided by the number of videos. With food: For each strain, videos (*n* ≥ 10 videos) collected from four independent experiments were analyzed. Without food: For each strain, videos (*n* ≥ 5 videos) collected from three independent experiments were analyzed. (D) Distribution of individual average speeds of detected tracks (*n* ≥ 487 tracks for each strain) with food (Day 1∼3 of adulthood). (E) Body length. Worms at 1 day of adulthood were used (*n* ≥ 113 for each strain). (F). Egg laying rate. Worms at 28 hr of adulthood were used. Shown is a combined result from two independent experiments with ∼10 hermaphrodites per well (*n* ≥ 12 wells for each strain). *p<0.01; **p<0.05; ***p<0.001; *p*-value by log rank test (Figure B) and t-test (Figure C, E, and F).

We conducted four different phenotypic assays using five different mutant strains involved in the Gαq signaling pathway (Figure 5): *egl-8*, *tax-6, eat-16*, *egl-30,* and *unc-73.*

In the lifespan assay, we found that *egl-8* and *tax-6* lived longer (Figure 5A and B), a finding consistent with previous reports [16], [25]. Both *eat-16* and *egl-30* mutants in our assay had shorter lifespans than N2. The reduced lifespan of *egl-30* under our QuantWorm system was inconsistent with other reports [16], [21], [26] and revealed the limitations of our system. Our system is locomotion based, thus, paralyzed animals would be classified as dead. Further, when worms move to the area near the wall on an agar plate, our system would not detect them and would count them as dead.

In the locomotion assay, *egl-8*, *egl-30*, *unc-73* and *tax-6* mutants had a lower mean velocity than N2 in the presence and absence of food while *eat-16* mutants had a higher mean velocity than N2 (Figure 5C). Our findings are consistent with results from other studies where *egl-30*, *egl-8* and *unc-73* mutants displayed lower body bend rates than N2 [17], [18], [20], [27] and *eat-16* mutant showed an increased number of body bends [18], [19], [28]. The result is also consistent with our previous report using a different single-worm tracking system [21]. Our measurement of N2 speed was within the ranges reported by multiple labs (31∼120 µm/s with food and 16∼250 µm/s without food, summarized by Ramot et al. [7]). Variation in N2 worm speed between papers is likely due to different worm ages or experimental conditions. We also observed different patterns in the speed probability distribution between strains. For example, compared with N2 and *egl-8, eat-16* had a broad spectrum of worm speed through 0–250 µm/s, meaning greater variance of worm speed (Figure 5D).

In the body size assay, we found that both *eat-16* and *tax-6* mutants had significantly shorter body length (t-test, p<0.001) whereas the *egl-30* mutant had a longer body size (Figure 5C). The body length of the *tax-6* mutant (795±71 µm) was about 70% of that of N2 (1126±77 µm), which is similar to the 60% change reported by Morck and Pilon [29], although their worm age (2 days after L4 larval stage) was different from ours (1 day after L4 larval stage).

We also assayed egg laying rate (Figure 5E). The measured egg laying rate of N2 worms in our study (5.85±1.33 eggs/worm/hr at 28 hr of adulthood) was similar to that reported by Daniels et al. (∼6 eggs/worm/hr) [30]. Compared with the egg laying rate of N2 worms, *egl-8*, *egl-30*, *unc-73*, and *tax-6* mutants had lower egg laying rates at 28 hr of adulthood. However, the reduction in the egg laying rate of *egl-8*, *egl-30*, *unc-73*, and *tax-6* mutants was much greater than that of *eat-16*.

## Discussion

The QuantWorm hardware configuration uses a microscope with a digital camera. While a flat-bed scanner could provide faster and cheaper imaging systems [1], [5], these systems have relatively lower image resolution. A flat-bed scanner is also incapable of creating high frame-rate video files, which are needed for advanced locomotion analysis. For lifespan assays, the plates are not accessible for manual observation if the system uses a flat-bed scanner [1], [5]. This limits its applications where the worms need to be dosed with a chemical and observed every day as in our first case study.

The QuantWorm software has adapted methods from several popular systems, so that the developers familiar with these systems easily modify QuantWorm. For example, while WormScanner provides a completely different user interface and functionality, it follows the same coding platform of Worm Tracker 2.0 (http://www.mrc-lmb.cam.ac.uk/wormtracker/). The WormLocomotion software uses an algorithm that is similar to the Parallel Worm Tracker [7]. However, WormLocomotion does not rely on the commercial software MATLAB. WormLocomotion also has enhanced functions in noise removal so that it has lower requirements for video quality. WormLocomotion only analyzes moving worms to prevent faulty analysis of worm-like objects that appear in video.

The QuantWorm system has several limitations in the lifespan assay, as illustrated in the case of the *egl-30* mutant lifespan. Our system detects moving worms and, therefore, measures healthy lifespan. If living worms remain stationary or move very slowly, the software may not detect these worms. Consequently, WormLifespan tends to count fewer worms than the traditional counting method based on touch-provoked movement with a platinum wire. Tapping the plate can agitate the animals and may mitigate such a problem. Further, when worms moved into the dark side of the well, they become no longer visible on the images. This problem is more severe when the animals are younger. Counting dead animals instead of live animals may alleviate the problem.

The accuracy of locomotion measurements by QuantWorm depends on the hardware setup. For example, in our settings, one pixel in video image corresponds to ∼20 µm. Therefore, we cannot accurately measure the speed of worms moving at a speed below this detection limit. This problem is partly shown in Figure 5C where *egl-8*, *egl-30*, *unc-73*, and *tax-6* had a similar saturated speed at ∼23 µm/s with food. Using a high-resolution camera or taking videos at a higher magnification can lower the detection limit.

Despite these limitations, the QuantWorm system proved to be a powerful tool as shown in our two case studies. In the first case study, we demonstrated that celastrol can significantly increase the lifespan. The time needed to analyze over 400 animals using the QuantWorm system was less than the time needed to assay less than 80 animals manually. Such throughput is highly desirable in chemical screens.

In our case study 2, we obtained consistent results for lifespan, locomotion, body length, and egg laying rate with those previously reported. The only exception was the *egl-30* mutant’s lifespan as previously discussed. It has been proposed that EAT-16 inhibits EGL-30 [19] and that EGL-30 activates both EGL-8 [27], [31] and UNC-73 [20] (for review, see [32], [33]). Consistent with this model, our data showed that the *eat-16* mutant had phenotypes opposite to that of EGL-8, EGL-30, and UNC-73 in lifespan, locomotion, and body length. It has also been proposed that TAX-6 activates EGL-30 [34]. Consistent with this model, we observed that TAX-6 and EGL-30 showed similar phenotypes in locomotion and egg-laying rates. However, TAX-6 and EGL-30 showed opposite phenotype in body length suggesting that TAX-6 may have different functions in body size regulation.

The QuantWorm system also enabled us to discover new phenotypes of these mutants. For example, it was known that *eat-16* animals move at a higher speed than wild-type animals on average, we also found that *eat-16* animals showed a broader variation in their speed distribution. It was reported that *eat-16* mutants lay premature eggs, while *egl-8* and *egl-30* mutants lay eggs that are at a later developmental stage than wild-type [35]. The QuantWorm system does not distinguish between the developmental stage of eggs, however, we found that *egl-8* and *egl-30* also lay eggs at a slower rate than wild-type.

The QuantWorm provides a powerful phenotyping tool that can be used in large-scale, quantitative genetic or chemical screens in *C. elegans*. As the functionality of the system is highly modular, it can easily accommodate additional analysis of new phenotypes. With additional image analysis tools, it can also be adapted to analyze other organisms.

## Supporting Information

Movie S1 **Worm simulation video mimicking sinusoidal movement of real worms moving forward.** The simulation video was computationally created to evaluate the accuracy of WormLocomotion. The video (7 frames/sec) contains 10 virtual worms moving at a velocity of 1 pixel/frame and 10 worms moving at a velocity of 0.25 pixels/frame.
(AVI)
